# Dangers of the Uterine Tampon1Read before the Central New York Medical Association, Buffalo, N. Y., October 16, 1894.

**Published:** 1895-05

**Authors:** Willis E. Ford

**Affiliations:** Utica, N. Y.


### DANGERS OF THE UTERINE TAMPON.[1]

By WILLIS E. FORD, M. D., Utica, N. Y.

The advent of a new method of treatment, which promises to speedily accomplish results that have heretofore been obtained slowly, if at all, naturally is accepted with enthusiasm. If we add to this the statement that the new method is devoid of danger, the chances are that it will be tried by a great number of physicians, and that the cases will not always be carefully selected. It is some time since the vigorous curetting and packing with gauze of a diseased uterus has been pretty generally practised, and enough cases are before us to make a general survey of this method of treatment profitable. That it is a quick and brilliant mode of reaching results in certain cases, is on all hands agreed; but that it is beneficent in all cases, as it is now being applied, is very doubtful. It is so easy, however, to adversely criticise almost any legitimate surgical procedure, that at the outset I desire to disclaim any intention of detracting from its real merits, and only aim to point out some dangers that my experience has thrust upon me.

The dull curette has for years been successfully used where there were villosities or polypoid masses or retained fragments of placenta to be removed, and if the operators had gone a little further, and had insisted on the dilatation of narrowed cervices and subsequent attention to the proper drainage of the uterus, there would not have been the necessity for so radical a change in methods as has lately been seen.

The sharp curette, or spoon, has for a long time been necessary for the removal of sloughing cancerous masses, or for fibroid nodules, but not until quite recently has it been advocated for endometritis. Topical applications, which have in the main, and justly, been discarded, never accomplished what in favorable cases the curette and the tampon is seen every day to accomplish. The danger of adopting any really valuable treatment as a cure-all is, however, apparent, and the danger in certain cases of the curette and uterine tampon ought not to be overlooked. Endometritis is such a stubborn disorder that vigorous measures, amounting to a surgical operation, are justifiable; but there are certain conditions that preclude mechanical violence, even in the name of surgery. Such are the cases of fixed malpositions, due to adhesions from inflammatory exudates about the adnexa; and such, also, it seems

1. Read before the Central New York Medical Association, Buffalo, N. Y., October 16, 1894.

to me, all those cases of catarrhal salpingitis accompanying endometritis. Those who first advocated the sharp curette after thorough dilatation of the entire cervical canal and of the body of the uterus, when necessary, did not advocate the tampon of gauze; and several men who are entitled to speak authoritatively on such matters, notably W. Gill Wylie, in this state, preferred other means to secure drainage—either a hard rubber instrument or open wire stem, or they left the dilated uterus to drain itself. Some critics, notably the Prices, of Philadelphia, did not approve the violent dilatation necessary, nor the device for drainage. The assertion was made that the theory was wrong, and that the injury to the uterine mucosa was found to set up salpingitis in a considerable number of cases, and that the last end of these patients was worse than the first.

It now seems to me that these differences of opinion were due to the fact that cases of perimetritis were accepted without previous treatment to release adhesions, and chiefly, perhaps, to the manner in which the gauze tampon was made.

On the whole, however, the results have been so good that now not only gynecologists, but general practitioners, at least in Central New York, are practising rapid dilatation, curettement more or less vigorous, and tamponing with gauze more or less firmly in a large number of cases. I am aware, however, that some deaths have followed this procedure, and that many cases of salpingitis are found whose history would seem to point to the operation as a cause. Last year I repaired a perineum for a woman living in a distant part of the state, and knew from her history and from careful examination that she had no tubal disease. Some six months afterward, her physician wrote me that she had just consulted him for great pain following a tamponment of the uterus for some catarrhal trouble, and he found in the left broad ligament a tumor larger than a hen's egg, very sensitive but entirely movable. While manipulating it, the sac ruptured. He was much alarmed and put the woman in bed, and after a short and not serious illness, she recovered sufficiently to be sent again to St. Luke's Hospital. I found a thickened tube and the uterus retracted, and moderately fixed to the left.

Another case, not so fortunate in its outcome, followed dilatation and tight tamponing of the uterus for dysmenorrhea. She was about twenty years old and unmarried. Her physician, an excellent general practitioner, said he was careful as to sepsis, but that twenty-four hours afterward she had a chill and rise of temperature. Soon after the gauze was removed, salpingitis followed, and two months later, when I saw her, she had on the right side an encysted pyosalpinx, holding more than a pint of pus, and was so feeble from sepsis that her friends declined to have any operation done. Such cases, I presume, are familiar to most of you who practise gynecology, and I need not take your time to relate more specific instances of what I believe to be due to the accident of too tight tamponing, especially of the cervical canal.

Many of my cases have been in the practice of such excellent surgeons, I cannot think the accident due to want of attention to antiseptic precautions, and while divulsion of a cervix or the most gentle scraping of an endometrium may occasionally be followed by considerable disturbance, the result is not generally an encysted salpingitis. Therefore, I do not think the accident the result of the dilatation nor of infection at the time of the operation, but a careful examination of the histories of more than a dozen such cases points to the tampon as the cause of tubal disease.

The original idea that gauze was necessary to keep the neck of the uterus open after thorough dilatation, as well as to drain the uterus, was manifestly based upon two errors. First, the uterine canal does not close down and prevent drainage after such operation; and secondly, iodoform gauze is not a drainage-tube, and does not remove the pus that may ooze into it. The statement that the gauze tightly packed in the cavity of the uterus after curettement causes a kind of involution, or that it improves the circulation of the parts, is not affirmatively proven by the cases I have seen, while often the immediate effect of dilatation, curettement with a dull instrument and thorough swabbing with carbolic acid or chloride of zinc, is followed by loss of bulk, reduction of tumefaction, and, it seems to me, the consequent improvement of circulation without tamponing. Of course it is understood that as perfect drainage of the uterus as it is possible to secure is aimed at always. If more attention was given to maintaining a proper position of the organ after curettement, and less reliance placed upon gauze or any other device to secure drainage, better results would be obtained and fewer accidents occur. All must have observed after curettement and packing that when the gauze is removed, the last layers contain some detritus and often pus, which has not been drained, but retained. Iodoform gauze is not an antiseptic, and within the uterus allows only the bloody serum to escape, and retards putrefactive changes, but does not remove or sterilize pus that comes down from a diseased tube. In purulent endometritis, therefore, with the organ movable and in normal position, the gauze tampon is not necessary. If the uterus is fixed even slightly, it is dangerous, because of the probability of catarrhal salpingitis being present, and the danger increases in proportion to the firmness of the tampon. If the uterus is fixed in a malposition, means ought first to be employed to release the adhesions and to restore the organ to as nearly a normal position as possible before curettement, and no gauze, or but a very light tampon of it, ought then to be applied.

Among the agents to be employed to release adhesions, and to thus pave the way for proper drainage, I would place galvanism at the head of the list, and for those who do not use this, the hot vaginal douche, vaginal tampons of boro-glyceride, iodine, etc. The first serious accident from curettement and packing I ever saw was in a case with adhesions, and the woman was very ill three months, and got up with double salpingitis.

In cases of hemorrhagic endometritis without polypoid growths or villossities, the good results attributed to the sharp curette and packing are more frequently due to the thorough dilatation, and to the topical application of a strong antiseptic, than to any other thing. I have often failed to cure these cases with the curette and tampon, and have obtained better results from thorough dilatation and topical applications, especially in young women.

The tight tampon of iodoform gauze is necessary often to stop hemorrhage, but in this case I remove it in thirty-six hours.

I have several times succeeded in draining through the uterus an encysted salpingitis, where the uterus and tumor were movable, by full dilatation and very tight tamponing. In these cases, the tube was not folded on itself and displaced, but was spindle-shaped and in nearly the normal position. There the tight tampon seems to me to be called for, but this interesting subject is not within the proper limits of this paper.

DISCUSSION.

Dr. H. T. Williams: I think the paper leaves nothing to discuss, Mr. President, the author having gone over the ground so thoroughly, and I simply wish to endorse the paper. I think that a paper like that does a great deal of good, especially to the general practitioner. I have seen a great deal of harm done by tamponing. My experience is it is not necessary to apply a tight tampon. A simple strip of gauze will frequently stop the hemorrhage without any pressure or force used at all. Another important thing after curetting the uterus, is to irrigate the uterus with mild antiseptics or water, and not use too much force in the irrigation, and to use hot water after that. There are very few cases in my experience that require tamponing at all.

